# A multi-study examination of the role of repeated spaced retrieval in the word learning of children with developmental language disorder

**DOI:** 10.1186/s11689-021-09368-z

**Published:** 2021-05-15

**Authors:** Laurence B. Leonard, Sharon L. Christ, Patricia Deevy, Jeffrey D. Karpicke, Christine Weber, Eileen Haebig, Justin B. Kueser, Sofía Souto, Windi Krok

**Affiliations:** 1 Department of Speech, Language, and Hearing Sciences, Purdue University, West Lafayette, IN USA; 2 Department of Human Development and Family Studies and Department of Statistics, Purdue University, West Lafayette, IN USA; 3 Department of Psychological Sciences, Purdue University, West Lafayette, IN USA; 4 Department of Communication Sciences and Disorders, Louisiana State University, Baton Rouge, LA USA; 5 Department of Communication Sciences and Disorders, Butler University, Indianapolis, IN USA; 6 Department of Speech and Hearing Science, University of Illinois Urbana-Champaign, Champaign, IL USA

**Keywords:** Developmental language disorder, Specific language impairment, Retrieval, Word learning, Language development

## Abstract

**Background:**

Many children with developmental language disorders (DLD) have well-documented weaknesses in vocabulary. In recent years, investigators have explored the nature of these weaknesses through the use of novel word learning paradigms. These studies have begun to uncover specific areas of difficulty and have provided hints about possible intervention strategies that might help these children learn words more accurately and efficiently. Among the studies of this type are those that incorporate repeated spaced retrieval activities in the learning procedures.

**Methods:**

In this study, we examined the data from four of these studies that employed the same types of participants (4- and 5-year-old children with DLD and same-age children with typical language development), research design, and outcome measures. The studies differed primarily in the type of learning condition that was being compared to a spaced retrieval condition. A mixed-effects modeling framework was used, enabling the data from the four studies and different outcome measures to be aggregated.

**Results:**

Across the studies, more words in the repeated spaced retrieval condition were recalled than those in the comparison conditions. This was true regardless of outcome measure. Children with typical language development recalled more words than the children with DLD. Both groups benefited from spaced retrieval, though effects were larger for the group with DLD. Children recalled words as accurately 1 week after learning as they did at the 5-min mark; the two groups were essentially identical in this respect.

**Conclusions:**

Overall, the findings support the continued refinement of these types of repeated spaced retrieval procedures, as they may have potential to serve as effective approaches to intervention.

**Supplementary Information:**

The online version contains supplementary material available at 10.1186/s11689-021-09368-z.

## Background

A longstanding finding in the memory literature is that when learners regularly test their recall of information during the learning process, their retention of the information improves [1–4]. Implied in this observation is that instead of being only a measure of what has already been learned, testing appears to create learning. This insight has been the impetus for numerous studies over the years, with a resurgence in this line of research in the last 15 years (see reviews in [5–8]). In this paper, we examine this issue as it relates to children’s word learning. We focus on one group of children at risk for word learning difficulties — children with developmental language disorder (DLD).

DLD is a life-long neurodevelopmental disorder whose most prominent symptom is a significant deficit in language ability [9, 10]. Individuals with DLD exhibit normal hearing, they show no evidence of neurological damage or disease, and they do not display the behavioral symptoms indicative of autism spectrum disorder. As a group, these individuals score slightly below their age mates from the same community on tests of nonverbal intelligence, yet their scores are reliably above the level of intellectual disability.

Although the term “developmental language disorder” can be found in the literature at least as early as 1961 [11], it has only recently become preferred over the term “specific language impairment” [12]. The latter term has often been applied to children with language disorders meeting a somewhat narrower set of criteria, such as earning a nonverbal intelligence score of 85 or above. Given current use, children meeting the criteria for specific language impairment also meet the broader set of criteria for DLD [9].

For many cases of DLD, there is a clear genetic contribution, yet, to date, studies have not yet identified any single gene whose variant or mutation could explain the disorder. The causes of DLD appear to be multifactorial. Weaknesses that extend beyond language can be seen in some individuals with DLD, yet these weaknesses cannot account for the language disorder itself [12]. Although increasing research attention has been directed toward adults with DLD, the bulk of research on DLD has focused on children.

Children with DLD are often described as showing deficits in morphosyntax, but weaknesses in vocabulary are also very common (see reviews in [13, 14]). These children know fewer words than their peers and, of the words they do know, their understanding tends to be more superficial [15]. This weakness is not limited to the younger ages; the vocabulary ability gap between individuals with DLD and peers with typical language development becomes wider over time [16].

To better understand the dynamic nature of vocabulary ability in children with DLD, many researchers have employed a paradigm in which they ask children to learn a set of novel words. The number of exposures of each word and the contexts in which the words are presented are carefully controlled. Findings from these studies indicate that children with DLD require more exposures to each word to reach the same learning criterion level as their peers [17–21]. This is true whether the criterion is defined as successful comprehension of the novel word or successful production [14].

Most of the research on novel word learning in children with DLD has focused on factors such as the frequency of input provided, whether semantic embellishments are included, and the type of lexical class (e.g., nouns, verbs, adjectives) represented by the novel words [22–24]. Testing has usually occurred only at the end of the learning period, to determine how many words had been acquired. However, in recent years, several investigators have included testing trials during the course of learning. We refer to these testing trials as “retrieval trials” here, in keeping with the extant literature. In this “Background” section, we briefly review these retrieval studies, including our own work. We then describe a model extension study that aggregates the data from our studies using a mixed-effects modeling framework to draw conclusions about the broader effects of retrieval on the word learning of children with DLD.

### Why retrieval?

The literature on memory often refers to three processes — encoding, consolidation, and retrieval [25, 26]. Encoding refers to the formation of a representation of the item in memory. In word learning experiments, encoding is seen when learners are engaged in studying a list of words. Consolidation refers to the more gradual process of integrating encoded information with other information in memory, a process that aids long-term retention. Sleep appears to significantly facilitate the consolidation process [27]. Retrieval — our main focus here — is the act of calling up information from memory.

Retrieval is important because it can provide benefits to learning that go beyond that which occurs with study alone [28]. These benefits come in two forms. First, retrieval seems to promote even more effective encoding during study (e.g., [29]). This is seen when learners are given a pre-test of, say, word pairs (e.g., *tide–?*) before they have even studied the material. Then, following a study period (e.g., studying *tide–beach*), the learners are given a post-test identical to the pre-test. Learners who take the pre-test remember much more than learners who study the word pairs without taking the pre-test. It appears that the retrieval attempts occurring during the pre-test set the stage for more successful encoding during the study period.

The second type of benefit provided by retrieval is a significant boost to long-term retention. This is seen in its simplest form when two learning conditions are equivalent in amount of study time but one condition also includes retrieval practice; the latter tends to produce better long-term recall [8]. However, the long-term recall advantages of retrieval are even greater with two kinds of manipulations. The first is the frequency of retrieval opportunities provided during study. When all else is equal, more attempts at retrieval result in greater recall [30]. The second is the spacing of retrieval attempts. Spacing can be defined in terms of time but also in terms of intervening material. Ideal spacing is one which is “effortful” but short enough to prevent forgetting. Repeated retrieval that occurs immediately after study with no intervening material can give the impression of being effective because it is often successful in the moment. However, it does not yield the long-term recall seen for repeated spaced retrieval [31].

Regardless of the frequency and spacing of retrieval, feedback following a retrieval attempt further assists long-term recall. In many studies, this feedback takes the form of the correct answer being provided after the learner’s response. Not surprisingly, such feedback aids subsequent retrieval success when the learner’s initial recall attempt is inaccurate or the learner offers no response. Just as importantly, such feedback is also effective even if the learner’s initial retrieval attempt is correct [32]. This is especially true when learners are not confident that their response is, in fact, correct.

### Retrieval and word learning in individuals with DLD

Thanks in part to the recent resurgence in retrieval-based research, several researchers have begun to explore the role that retrieval might play in the word learning of children and young adults with DLD [33–35]. The McGregor et al. study [35] included comparisons between retrieval and no-retrieval learning conditions. Words that included retrieval opportunities during the learning period had better recall 1 day later than words limited to study during the same period. In the more recent McGregor et al. study [34], all words included retrieval trials. The words were presented until the young adults reached a predetermined level of accuracy. Once this criterion level was reached, the words were divided according to the timing of a recall test. One third of the words was tested 1 day after the learning period, another one third was tested 1 week later, and at the one-month mark, all words were tested (two thirds for a second time). The adults with DLD required more retrieval practice than a group of typical language peers to reach the criterion level. Although they were not as successful as their peers at the 1-week testing, their recall matched the peers both at 1 day and 1 month. McGregor et al. also found that the recall test at 1 week was significantly beneficial to performance at 1 month.

Our research group has conducted a series of four studies, all with the purpose of evaluating the effects of repeated spaced retrieval on the novel word learning of 4- and 5-year-old children with DLD and their same-age peers with typical language development (TD). The results of the individual studies have been reported elsewhere (see below). In this paper, we present analyses of the results that cross the four studies and the types of tests used to assess recall and recognition of the novel words. By using the aggregated data, we are able to evaluate the robustness of repeated spaced retrieval benefits across different comparison conditions, slight variations of spaced retrieval schedules, and different outcome measures. The similar designs and measures across the studies allowed for pooled analyses via a mixed-effects modeling framework akin to a traditional meta-analysis, but evaluating direct observations rather than published study estimates. Specifically, we ask whether (1) across studies, repeated spaced retrieval holds a learning advantage over a variety of comparison learning conditions; (2) children with DLD benefit from repeated spaced retrieval as much as or more than children with TD; (3) any benefits seen from repeated spaced retrieval are still evident 1 week after the learning period; and (4) the advantages of repeated spaced retrieval can be seen in different types of outcome measures (word form recall, meaning recall, recognition).

## Method

### General design of the four studies

The four studies were (1) Leonard et al. [36], (2) Haebig et al. [37], (3) Leonard et al. [38], and (4) Leonard et al. [39]. These studies will be referred to as studies 1-4, respectively. The study by Haebig et al. [37] (study 2) also included electrophysiological (ERP) measures; these are not examined here, although the results were consistent with the results that we do report in this analysis.

The overall design was the same for all four studies. The key distinctions between the studies are summarized in Table 1; these are discussed in more detail in the following relevant subsections. For each study, a within-participant design was used. Children with DLD and children with TD learned the novel words in two sets, with each set studied and tested over 2 weeks, in succession. The words were divided into two sets (separated by a week) out of concern that a single, longer set of words might result in low recall scores even by children with TD. In each set, half of the words were presented in a repeated spaced retrieval condition and half were presented in a comparison condition, with the words assigned to each condition counterbalanced across the children in each group. The novel words and their referents were presented to the child in individual sessions via laptop computer. The experimenter sat next to the child and controlled the pace of the presentation. For each set, there were two learning sessions held on consecutive days. Each session was approximately 20 min in duration.

**Table 1:** Features of study design

Study	RSR^a^ condition	Comparison condition	Novel words	Referent type	Test types
1	033^b^	RS^c^	/dɔik/, /pαɪb/, /gɪf/, /nɛp/, /fαʊn/, /jʌt/, /bog/	Noun	Word form, meaning, recognition
2	022^d^	IR^e^	/bog/, /nɛp/, /paɪb/, /jʌt/, /daɪbo/, /fumi/, /gine/, /tomə/, /kodəm/, /meləp/, /pobɪk/, /tɛkət/	Noun	Word form, meaning, recognition^f^
3	033	RS	/fɪm/, /taɪmɪk/, /zogi/, /beɪp/, /næfi/, /mok/, /kudɪp/, /paɪt/	Adjective	Word form, recognition^f^
4	More retrieval/less study	More study/less retrieval	/fumi/, /jʌt/, /nɛp/, /tɛkət/, /bog/, /paɪb/	Noun	Word form, meaning, recognition^f^

^a^Repeated spaced retrieval

^b^Spacing with 0 intervening words and then 3 intervening words

^c^Repeated study

^d^Spacing with 0 intervening words and then 2 intervening words

^e^Immediate retrieval

^f^Recognition tested at 1 week only

In studies 1–3, the words in the two conditions were presented the same number of times. (In study 4, degree of exposure served as an independent variable.) In the first three studies, retrieval trials were followed by study trials which enabled the children to hear the words they just attempted to retrieve, thus representing a type of feedback. This occurred regardless of the accuracy of the children’s preceding retrieval attempt. The children were not told if their response was correct.

Five minutes after the second session, recall tests were administered. These tests were repeated 1 week later, along with a recognition test. These tests were also presented via laptop computer, controlled by the experimenter. The children’s responses on the word form tests (testing the child’s recall of novel words such as /bog/ and /kudɪp/) were scored as correct or incorrect using an adaptation of a system developed by Edwards et al. [40]. In this system, each consonant produced is credited with one point each for correct place, manner, and voicing. Each vowel produced is given one point each for correct backness, height, and length. An extra point is awarded for correct syllable shape (e.g., consonant-vowel-consonant). A production was scored as correct if it appeared subjectively as an attempt at the correct word and if the point total earned was higher than the point total assigned if the production was assumed to be an attempt at a different novel word. This scoring method allowed for phonetic imprecision while significantly reducing the likelihood that the child was attempting a different novel word.

In each study, the data analysis involved a between-participant comparison (DLD vs TD), and two within-participant comparisons — a learning condition comparison (repeated spaced retrieval vs a comparison condition) and a time comparison (testing 5 min after learning vs 1 week later). Within each study, separate analyses were conducted for each type of test administered (word form recall, meaning recall, recognition).

### Participants

Table 2 provides a summary of the participant characteristics in the four studies. Both the children with DLD and their peers with TD ranged in age from 4;0 (years;months) to 5;11. All children passed a hearing screening and had no history of neurological damage or disease. All children scored well above the level of intellectual disability on the Kaufman Assessment Battery for Children–Second Edition (KABC-2 [41]) or the Primary Test of Nonverbal Intelligence (PTONI [42]). In keeping with current nonverbal intelligence criteria for DLD, we required a score above 75 for inclusion — a score above the level of intellectual disability even after the test’s standard error of measurement is taken into consideration (see [12]). However, in practice, all but three children had scores of 85 or above that match the more stringent criterion used in many studies of children described as exhibiting specific language impairment. The remaining children — all in the DLD group — had scores of 81–83 (2 in study 3 and 1 in study 4).

**Table 2:** Summary of participant characteristics

Study	Test/measure	Group	
1		DLD (*n* = 10)	TD (*n* = 10)
	Age	63.40 (6.20)	63.20 (4.89)
	Maternal education	15.10 (2.23)	16.90 (2.56)
	SPELT-P2	74.70 (12.48)	118.90 (7.48)
	K-ABC2 or PTONI	108.40 (12.14)	121.60 (17.06)
	PPVT-4	97.67 (9.70)	115.20 (13.21)
2		DLD (*n* = 16)	TD (*n* = 16)
	Age	59.60 (4.43)	61.58 (5.16)
	Maternal education	15.50 (1.59)	16.63 (1.75)
	SPELT-P2	78.69 (9.41)	113.06 (9.17)
	K-ABC2	101.88 (8.00)	115.81 (10.06)
	PPVT-4	103.44 (9.91)	121.06 (12.47)
3		DLD (*n* = 14)	TD (*n* = 13)
	Age	62.64 (5.41)	62.54 (6.34)
	Maternal education	14.79 (2.19)	16.69 (1.65)
	SPELT-P2	76.93 (15.78)	119.00 (8.03)
	K-ABC2	99.21 (12.88)	114.31 (11.06)
	PPVT-4	102.57 (11.33)	118.62 (13.62)
4		DLD (*n* = 13)	TD (*n* = 13)
	Age	56.69 (6.50)	57.80 (6.47)
	Maternal education	16.54 (2.67)	16.15 (2.34)
	SPELT-P2	77.15 (11.89)	113.46 (11.11)
	K-ABC2	103.62 (13.52)	112.00 (8.51)
	PPVT-4	103.77 (13.64)	121.85 (7.26)

Mean chronological age and maternal education level in years (and standard deviations) and mean standard scores (and standard deviations) on the standardized tests administered to the children with developmental language disorder and the children with typical language development. One child in the DLD group in study 1 was not administered the Peabody Picture Vocabulary Test–Fourth Edition (PPVT-4).

The children with DLD were enrolled in language treatment or were scheduled to be enrolled. In addition, we applied a two-step process for selection to the DLD group. First, we determined if the child’s standard score was below 87 on the Structured Photographic Expressive Language Test–Preschool 2 (SPELT-P2 [43]), the level showing acceptable sensitivity and specificity reported by Greenslade et al. [44]. If a child’s score fell just above the cut-off, the child was required to score below the sensitivity/specificity cut-off on the finite verb morphology composite (FVMC [45]) or below the 10th percentile on Developmental Sentence Scoring (DSS [46]). Seven children qualified for the DLD group based on the FVMC/DSS; the remaining children qualified on the basis of the SPELT-P2 scores. All children in the DLD group scored in the “Minimal to No Symptoms of ASD” range on the Childhood Autism Rating Scale–Second Edition (CARS-2 [47]). Tests were also administered for strictly descriptive purposes; these differed somewhat depending on the individual study in which a child participated. The PPVT-4 [48] was administered to all children. As a group, the children with DLD scored in an age-appropriate range on this test, though well below the TD children in each study serving in the comparison groups. This is a frequent finding in the DLD literature (e.g., [49]), owing possibly to the fact that vocabulary tests have inadequate sensitivity and specificity [50, 51].

The children with TD were very similar in age to the children with DLD. All met the selection criterion for the TD group by scoring above the cut-off score for the SPELT-P2. They also scored higher than the DLD group on the KABC-II and the PPVT-4. These children were not administered the CARS-2, as, during the prescreening process, parents expressed having no concerns about their child’s language or cognitive development.

### Study 1

This study was a comparison of a repeated spaced retrieval (RSR) learning condition and a repeated study (RS) learning condition. The children were asked to learn eight novel monosyllabic words (e.g., /bog/) that served as nouns referring to exotic plants and animals shown in photographs. We refer to these novel nouns as “word forms.” The children also learned what each plant or animal “liked” (e.g., snow). We refer to these as “meanings.” Within each set, the words were presented in alternating order (e.g., a word in the RSR condition followed by a word in the RS condition), with the order counterbalanced across children. There were 16 study trials for each word, regardless of learning condition. For each study trial, the child saw the picture on the laptop and heard (using /bog/ as the example), “This is a /bog/. It’s a /bog/. A /bog/ likes snow.” For each word in the RSR condition, there were also 12 retrieval trials. In these trials, the child saw the picture and was asked “What’s this called? What do we call this?” Responses to this type of question assessed the child’s retrieval of the word form. The child was then asked “And what does this one like? What does it like?” to assess success in retrieving the meaning. Note that although the “meaning” assigned to each referent was arbitrary, the retrieval of meanings was likely inherently easier than the retrieval of word forms. For meaning, the children had to remember a familiar word (e.g., “snow”) that was associated with a visual referent. Recall of the name of the referent (e.g., /bog/) was not required.

**Fig. 1:**
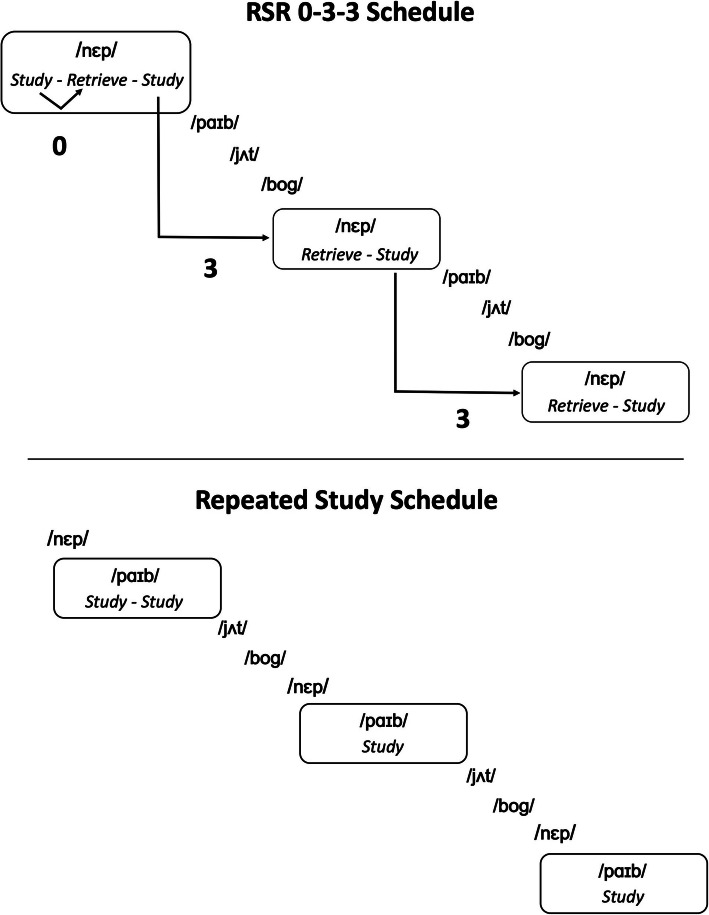
Examples of the first block of the learning period in Leonard et al. [36]. The top panel is an example of a novel word (/nɛp/) in the repeated spaced retrieval (RSR) condition. This novel word begins with a study trial followed immediately by a retrieval trial and then another study trial. The designation “0” indicates that there were zero words intervening between the retrieval trial and the preceding study trial. When (/nɛp/) appeared again in the learning sequence, it was in a spaced retrieval trial, with three other words intervening since the previous study trial for /nɛp/. For this reason, it is designated a “3” trial. The bottom panel shows a novel word (/paɪb/) in the repeated study condition. Only study trials were used in this condition. The number of study trials matched the number of study trials used in the RSR condition to control for amount of exposure

For words in the RSR condition, we employed what we refer to as a “0-3-3” schedule. An illustration is provided in Fig. 1. The word first appeared in a study trial and was then followed immediately by a retrieval trial, with no intervening words. The “0” refers to the fact that there were no other words intervening between the word’s previous study trial and the same word’s retrieval trial. Another study trial followed the retrieval trial (thus, study–retrieval–study). Thereafter, for each word in the RSR condition, there were three intervening words between the word’s previous study trial and its retrieval. These retrieval trials in the “3” phase are spaced retrieval trials. Each spaced retrieval trial was immediately followed by a study trial for the same word (thus, retrieval–study).

Words in the RS condition alternated with those in the RSR condition (see Fig. 1). Because the initial “0” phase in the RSR condition had two study trials (study–retrieval–study), two consecutive study trials (study–study) were initially used for words in the RS condition to ensure that the number of times each word was heard was the same for the words in the two learning conditions. Thereafter, only one study trial was used each time a word in the RS condition appeared in the learning sequence.

In total, each word form was heard 48 times, as a result of having 16 study trials with the word heard three times on each study trial (“This is a /bog/. It’s a /bog/. A /bog/ likes snow.”) Each meaning was heard 16 times, because meanings (e.g., snow) were presented only once during each study trial. The two conditions differed because the RSR condition also included 12 retrieval trials.

Five minutes following the second learning session, a recall test was administered to assess the children’s retention of the word form and meaning of each word. These tests were identical to the retrieval trials in their video and audio presentation. Each word form and meaning was tested twice; items testing the same form or meaning never appeared consecutively. A recognition test was then administered. For each item, the child saw three pictures on the laptop screen and heard the request to point to the correct picture after hearing the referent’s name (e.g., “Where’s the /bog/?”). Success on this test required the child’s recognition of the word form and its association with a particular visual referent. One week later, the identical tests were re-administered.

### Study 2

Study 2 was designed to compare a RSR condition with an immediate retrieval (IR) condition, asking whether it is the spacing of retrieval practice, or just any opportunity for retrieval practice that is beneficial to learning. As in study 1, the words in the two conditions were identical in the number of times they were heard by the child (24). However, in study 2, the words in the two conditions were also the same in the number of retrieval trials used (6). The difference is that in the IR condition, all retrieval trials were “0” trials. For the RSR condition, we employed a 0–2–2 schedule. Fewer intervening words were used in this study than in study 1, in part because we increased the number of novel words to be learned from eight in study 1 to 12 novel words in study 2. Four were monosyllabic (e.g., /jʌt/) and eight were disyllabic (e.g., /pobɪk/). Again, the novel words referred to exotic plants and animals. Both word forms and meanings were presented and later tested. Testing occurred 5 min after the second learning session and 1 week later. However, the recognition test was administered only at the 1-week mark and the child had to choose from among four alternative pictures on the laptop screen instead of only three pictures as in study 1.

### Study 3

Study 3 had the same design as study 1 — comparing a RSR 0-3-3 condition and a RS condition. However, in study 3, the words were novel adjectives. Pictures were artist-rendered drawings of common objects with unusual characteristics (e.g., a toothbrush with multiple curls instead of a straight handle). Eight novel adjectives were used, four monosyllabic (e.g., /beɪp/) and four disyllabic (e.g., /taɪmɪk/). To promote the idea that the novel words referred to attributes, two exemplars were used for each novel word (e.g., a toothbrush and a pencil) during the learning session. Study trials used the phrasing “This pencil is /taɪmɪk/. It’s very /taɪmɪk/. This pencil is really /taɪmɪk/.” Only word form information (e.g., /taɪmɪk/) was targeted in this study. Retrieval trials used a carrier phrase format, as in “Tell me about the pencil. The pencil is very ____.” Across the two learning sessions, all words were heard an equal number of times (44); the two conditions differed because the RSR condition also included 12 retrieval trials.

Five minutes after the second learning session the word form recall test was administered, identical in format to the retrieval trials. However, along with testing each exemplar used during the learning session, we tested two additional unfamiliar exemplars for each word (e.g., a spoon and stem of a flower with the properties of /taɪmɪk/). Thus, each word form was tested with four test items, with each item using a different exemplar. For our analyses, the two unfamiliar exemplar items were combined with the exemplar items used during learning, as accuracy was identical for the two types. The word form recall test was repeated 1 week later, followed by the (four alternative) recognition test (e.g., “Show me the one that is /taɪmɪk.”) that also included the newly introduced exemplars.

### Study 4

In the fourth study, the children were asked to learn six novel words representing the names of exotic plants and animals. Four were monosyllabic (e.g., /nɛp/) and two were disyllabic (e.g., /fumi/). Study 4 differed from the other three studies in the kind of comparison conditions used. Specifically, the conditions differed in the *degree to which* repeated spaced retrieval trials were included, rather than *if* they were included. During the first phase of the learning period, all words were presented with opportunities for both immediate and spaced retrieval. After an initial immediate retrieval trial, each novel word appeared in a retrieval–study–retrieval sequence. The first retrieval trial in this sequence was a spaced retrieval trial because one to four other words intervened between the word’s retrieval trial and its previous study trial. The second retrieval trial in the sequence was an immediate retrieval trial because it was immediately preceded by a study trial for the same word. Once the children were successful on four consecutive immediate retrieval trials for each word, they proceeded to the next phase of the study. At this point, half of the words were assigned to a retrieval-only condition; study trials were no longer included. The other half of the words were assigned to a study-only condition, with retrieval trials no longer appearing. We refer to these conditions as more retrieval/less study and more study/less retrieval, respectively. In total, all words had at least 8 study trials, 7 immediate retrieval trials, and 6 spaced retrieval trials. The words in the two conditions differed due to an additional 9 retrieval (only) trials in the more retrieval/less study condition and an additional 9 study (only) trials in the more study/less retrieval condition.

Following the two learning sessions, recall tests and recognition tests were administered as in study 2. Because the children had spaced retrieval opportunities with all of the words prior to the words being moved to a retrieval-only or study-only phase, we used the number of words successfully retrieved on spaced retrieval trials in the earlier phase as a covariate.

### Analytic methods

Data from the four studies described above were aggregated. The studies had primarily independent samples. The outcome for all studies was the number of correct responses on the test. In study 4 only, there was an initial repeated spaced retrieval phase for all words prior to the words being separated into distinct learning conditions. As noted above, in the original study, we used the number of words correct during this initial phase as a covariate, as it represented a type of “practice” score. Separate practice scores were used for the words in the two conditions because the practice scores were not the same for all words; they varied even within the same child. With these practice scores as a covariate, we could better guard against words in one condition having a better outcome than words in the other condition simply by virtue of holding an initial advantage. Because the other studies did not employ a measure of this type, outcomes for study 4 were adjusted for the practice score where residuals were generated from a bivariate regression of the number of correct words regressed on the practice scores. The residuals were then rescaled to have the same mean and variance as the original outcome and these adjusted values are used as the outcome in the present analyses.

In each study, two groups of children (DLD and TD), two learning conditions (RSR and the comparison condition, referred to as the “other learning” condition, OL), and two time points (5 min and 1 week) were compared. However, for three of the studies, recognition was assessed only at the 1-week post-learning period time point.

In our earlier reports of each individual study, each test type (word form recall, meaning recall, recognition) was analyzed separately. In the present analysis, we pool across all four studies as well as the three test types within studies to obtain more generalized effect size estimates as well as to evaluate differences across studies (involving different OL conditions) and test type effects.

A mixed-effects modeling framework was used where repeated measures are nested within children. Models included a random intercept at the child level as well as random slopes for the learning condition and test type effects. The time slope (5 min versus 1 week) did not have significant between-child variability. The random effects are estimated using an independent covariance matrix, which excludes covariances among the random intercept and slopes. The four studies are distinguished using fixed effects with dummy indicators where between-study mean differences are captured in the study indicator coefficients. Study is largely a between-child variable. However, three children participated in two different studies. For those children, the tests of differences between the two studies they participated in are within-child tests. The nesting of measures within child across studies is accommodated using the child-level random intercept. This approach exerted a small influence on the results across the models but had the advantage of allowing us to use all of the observations in the studies.

Three covariate controls were included. These were PPVT-4 standard score, mother’s years of education, and the number of items included on the test. Each study tested a different number of words and, in some cases, different test types within a study varied in the number of items on the test. Therefore, to prevent confounding of the maximum number of possible correct responses from study and test type differences, this variable was included as a covariate. A detailed description of the general, main effects model is provided in equation (1) of Supplementary Materials.

The main effects model was expanded to test interactions between fixed effects in a series of models. First, two-way interactions between the three primary experimental variables were tested. These variables included participant group, learning condition, and time. Another model added the three-way interaction of these variables. These models correspond to the models that were estimated for the separate study publications. However, in the present study these models provide aggregated effect size estimates across the four studies and three test types as well as estimates of study and test type differences in correct responses, which were not previously evaluated.

Both raw effect sizes and partially standardized effect sizes are presented. Raw effects provide the estimated differences in the number of correct responses as a function of a unit change in independent variables. For the partially standardized effect, the outcome is standardized, so that the effects provide the standard deviation difference in the outcome as a function of a raw unit change in independent variables. The partially standardized effects are comparable to Cohen’s *d* effect size but are conditioned on model covariates. Restricted maximum likelihood (REML) estimation was used in Stata Statistical Software, version 15.1 [52].

In another set of analyses, we pooled across all four studies but evaluated outcomes for word recall, meaning recall, and recognition separately. Details of the main effects model are shown in equation (2) of Supplementary Materials. In these models, we obtained generalized effect size estimates for the three primary experimental variables as well as evaluated differences across studies. A mixed-effects modeling framework was again used where repeated measures are nested within children. The number of items assessed on the test was not included as an independent variable in these models due to the linear dependency this variable had with the study indicators within test types. Therefore, study differences are confounded with number of items tested for the raw coefficients. The partially standardized coefficients are therefore preferred for comparing effects sizes across the studies in the test type-specific models. The study using adjectives (study 3) examined recall of word forms only, so there are no meaning recall outcomes for this study. Because most studies tested recognition only at the 1-week point, there were too few observations for the 5-min time point (38 repeated observations) for the recognition outcome; therefore, interactions of study with time were not testable. The time effect for recognition models should also be interpreted with caution due to the limited observations at 5 min.

## Results

### Main effects and interactions among participant group, learning condition, and time

Table 3 provides the main effects with the covariate controls. Prior to the inclusion of the covariates, there were effects for participant group (b_std_ = − 0.24, *p* = 0.013) and learning condition (b_std_ = 0.35, *p* = 0.000) but not time. As can be seen in Table 3, effects were virtually unchanged by the addition of the PPVT-4 standard scores and mother’s education. (Indeed, neither of these covariates was even correlated with the outcome scores.) However, as expected, controlling for the number of items on the tests changed the differences between studies and the differences between test types in the number of correct responses. For each additional item tested, there was an associated 0.58 higher number of correct responses on average.

**Table 3:** Main effects model results — pooled over studies and testing type (*n* = 101, 960 repeated observations)

Fixed effects	b	95% CI		*p*-value	b_std_
Group (DLD vs. TD)	− 1.40	− 2.48	− 0.32	0.011	− 0.31
Condition (RSR vs. OL)	1.59	1.19	1.98	0.000	0.35
Time (1 week vs. 5 min)	− 0.05	− 0.28	0.18	0.652	− 0.01
Study 2 vs. study 1	− 2.89	− 4.06	− 1.71	0.000	− 0.64
Study 3 vs. study 1	− 2.20	− 3.38	− 1.01	0.000	− 0.49
Study 4 vs. study 1	− 0.04	− 1.25	1.17	0.951	− 0.01
Study 3 vs. study 2	0.69	− 0.45	1.83	0.236	0.15
Study 4 vs. study 2	2.85	1.53	4.16	0.000	0.63
Study 4 vs. study 3	2.16	0.61	3.71	0.006	0.48
Meaning vs. word form	4.43	3.84	5.01	0.000	0.98
Recognition vs. word form	5.80	5.25	6.36	0.000	1.28
Meaning vs. recognition	− 1.38	− 2.16	− 0.59	0.001	− 0.30
Covariates					
PPVT-4	− 0.02	− 0.05	0.02	0.429	0.00
Mother’s education	0.00	− 0.21	0.22	0.982	0.00
Number of words tested	0.58	0.44	0.71	0.000	0.13
Intercept	1.15	− 4.20	6.49		
Random effects	σ^2^	95% CI			
Condition	2.87	1.84	4.49		
Meaning	5.33	3.55	7.99		
Recognition	3.28	2.07	5.21		
Intercept	3.99	2.80	5.71		
Residual	2.77	2.44	3.14		

*b*_*std*_ outcome standardized across studies and testing type

**Fig. 2:**
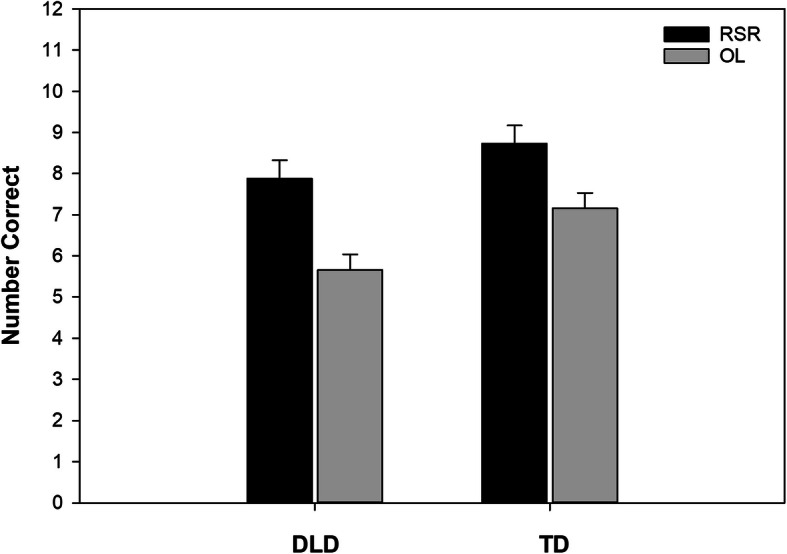
The marginal means showing differences according to participant group and learning condition (collapsed across time). DLD, children with developmental language disorder; TD, children with typical language development; RSR, repeated spaced retrieval condition; OL, other learning condition

As shown in Table 3, across studies and test types, the children with DLD had about 1.4 fewer correct responses relative to the TD children on average (b = − 1.40, *p* = 0.011). This represents nearly one third of a standard deviation lower value (b_std_ = − 0.31). The aggregated effect of the RSR conditions relative to the OL conditions was 1.59 more correct responses on average (*p* = 0.000). This represents over a third of a standard deviation benefit (b_std_ = 0.35). There was no difference in the number of correct responses between the 5-min post-learning period and 1 week later. The differences according to participant group and learning condition are illustrated in Fig. 2. Corresponding distributions are illustrated in Figure 1S in Supplementary Materials.

There were several study differences (Table 3). These differences were expected given that each study involved a different comparison condition, the retrieval schedules in the RSR conditions were not the same, and there were differences between the studies in the number of times each novel word was presented. There were also differences between each of the test types (Table 3). The highest scores were seen for recognition, followed by meaning, and then word form. (We explore later how participant group and learning condition effects were seen within each of these test types.)

The model reflecting two-way interactions appears in Table 1S in Supplementary Materials. The participant group-by-learning condition interaction did not meet an alpha level of 0.05, but it had the largest effect size (b = 0.65 and b_std_ = 0.14, *p* = 0.105). Examination of the simple effects (Table 4) reveals that the DLD group differed from the TD group in the OL conditions in particular, where the DLD group had 1.50 fewer correct responses (*p* = 0.009). There was an RSR advantage for both the DLD and TD group, but the effect was greater for the DLD group. The children with DLD had 2.22 more correct responses under the RSR condition relative to the OL conditions, on average, while the TD group had 1.57 more correct responses under the RSR condition (Table 4).

**Table 4:** Simple effects for the group by condition interaction

	b	95% CI		*p*-value	b_std_
DLD versus TD in RSR condition	− 0.85	− 2.14	0.45	0.201	− 0.19
DLD versus TD in OL condition	− 1.50	− 2.61	− 0.38	0.009	− 0.33
RSR versus OL condition for DLD group	2.22	1.61	2.84	0.000	0.49
RSR versus OL condition for TD group	1.57	0.96	2.18	0.000	0.35

*b*_*std*_ outcome standardized across studies and testing type

There was a learning condition-by-time interaction (b = − 0.52, b_std_ = − 0.11, *p* = 0.018). Although the RSR scores were higher than those of OL at both time points (see Table 2S in Supplementary Materials), the difference was greater at 5 min (b = 1.57, b_std_ = 0.35, *p* = 0.000) than it was at 1 week (b = 1.06, b_std_ = 0.23, *p* = 0.000). There was no participant group-by-time interaction; the children with DLD were indistinguishable from the TD group in their retention over the 1-week period (b = 0.00, b_std_ = 0.00, *p* = 0.991). Likewise, there was no three-way interaction among the participant group, learning condition, and time variables.

### Main effects and interactions for each test type

**Fig. 3:**
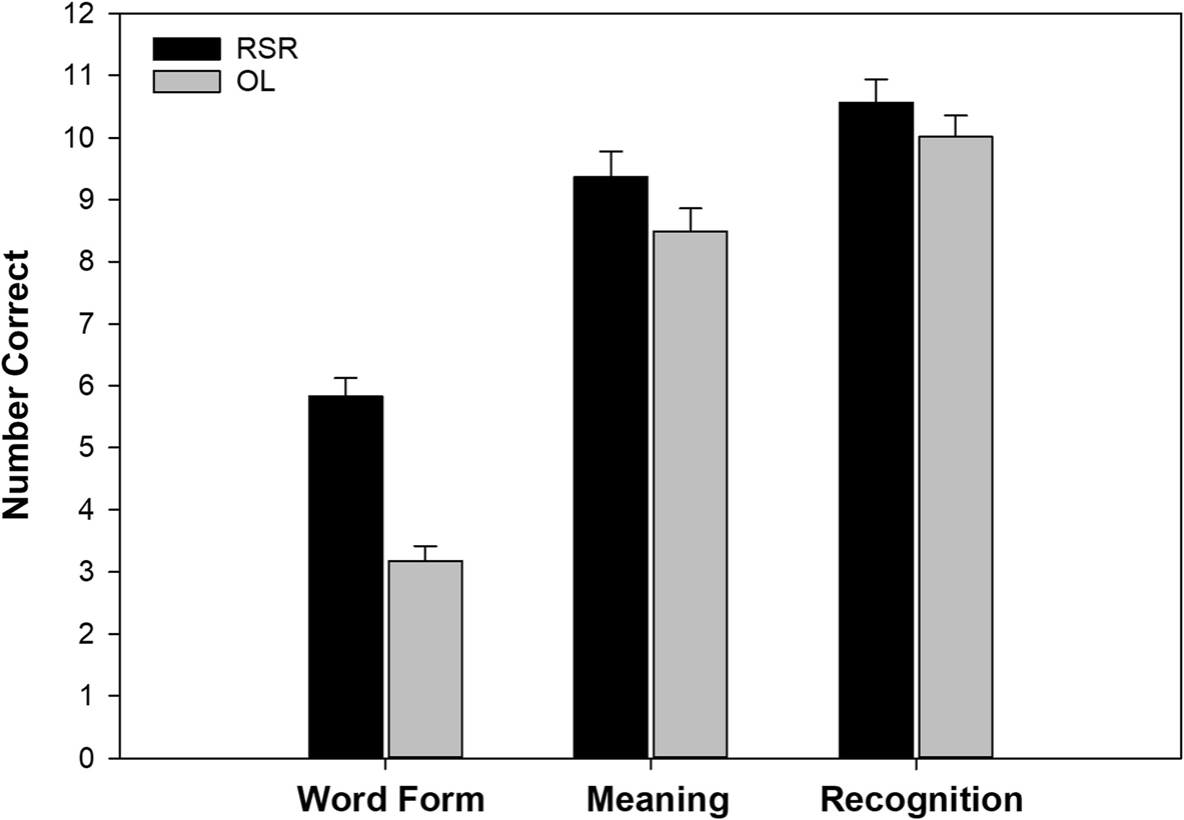
The marginal means showing differences according to learning condition across the three test types (collapsed across participant group and time). RSR, repeated spaced retrieval condition; OL, other learning condition

As noted in the “Analysis methods” section, in additional models, we pooled across all four studies but evaluated outcomes for word form recall, meaning recall, and recognition separately. An illustration of the findings appears in Fig. 3. Distributions are provided in Figure 2S in Supplementary Materials.

#### Word form recall

As shown in Table 5, across the four studies, children in the DLD group had 1.57 fewer correct responses relative to the TD group (b_std_ = − 0.44, *p* = 0.018). The RSR condition advantage over the OL conditions was 2.62 additional correct responses, on average, across the studies (b_std_ = 0.74, *p* = 0.000). There was no difference in correct responses between the 5-min and the 1-week post-learning period time points.

**Table 5:** Word form main effects model results — pooled over studies (*n* = 101, 416 repeated observations)

Fixed effects	b	95% CI		*p*-value	b_std_
Group (DLD vs. TD)	− 1.57	− 2.86	− 0.27	0.018	− 0.44
Condition (RSR vs. OL)	2.62	2.02	3.23	0.000	0.74
Time (1 week vs. 5 min)	0.01	− 0.24	0.26	0.948	0.00
Study 2 vs. study 1	− 0.11	− 1.45	1.23	0.872	− 0.03
Study 3 vs. study 1	3.22	2.22	4.21	0.000	0.91
Study 4 vs. study 1	1.00	− 0.29	2.29	0.127	0.28
Study 3 vs. study 2	3.33	2.11	4.55	0.000	0.94
Study 4 vs. study 2	1.11	− 0.17	2.40	0.089	0.31
Study 4 vs. study 3	− 2.21	− 3.31	− 1.12	0.000	− 0.63
Covariates					
PPVT-4	− 0.02	− 0.07	0.02	0.302	− 0.01
Mother’s education	0.01	− 0.25	0.27	0.949	0.00
Intercept	5.24	− 0.98	11.46		
Random effects	σ^2^	95% CI			
Condition	7.96	5.67	11.19		
Intercept	6.06	4.34	8.46		
Residual	1.70	1.39	2.09		

*b*_*std*_ outcome standardized across studies WITHIN testing type

The two-way interactions revealed no differences. From the simple effects for these models, both the DLD and the TD group showed clear effects favoring the RSR condition over the OL conditions (b = 3.13, b_std_ = 0.89, *p* = 0.000 and b = 2.50, b_std_ = 0.71, *p* = 0.000, respectively; Table 3S). The RSR condition produced higher scores than the OL conditions at both time periods (b = 2.50, b_std_ = 0.71, *p* = 0.000 and (b = 2.12, b_std_ = 0.60, *p* = 0.000; Table 4S). Scores were lower for the children with DLD than for the TD group at both 5 min (b = − 1.76, b_std_ = − 0.50, *p* = 0.009) and 1 week (b = − 1.48, b_std_ = − 0.42, *p* = 0.028; Table 5S). There was no three-way interaction between participant group, learning condition, and time.

#### Meaning recall

Across the studies, there was both a group effect and a learning condition effect for the meaning recall outcomes (Table 6S). Children with DLD had 1.25 fewer correct responses on average compared to the TD group (b_std_ = − 0.45, *p* = 0.027). The RSR learning condition resulted in 0.71 more correct responses relative to the OL conditions across the studies (b_std_ = 0.25, *p* = 0.001). There was no effect for time, and there were no two-way interactions or three-way interaction.

#### Recognition

Pooling across the four studies, there was both a participant group and learning condition effect for the recognition outcome (Table 7S). Children with DLD had 1.25 fewer correct responses relative to TD children (b_std_ = − 0.35, *p* = 0.011). The RSR learning condition resulted in 0.56 more correct responses on average compared to the OL learning conditions (b_std_ = 0.15, *p* = 0.003). There was no effect of time. Similarly, there were no two-way interactions or three-way interaction.

## Discussion

The mixed-effects modeling framework used in this study offered advantages over more traditional meta-analyses. This framework was facilitated by the essentially identical designs of the four studies. In all studies, the children with DLD and TD were of similar age, they learned novel words under two learning conditions, they were tested immediately after the learning period of the second day and then again 1 week later, and the three tests (word form recall, meaning recall, recognition) were of the same type in each study. Capitalizing on these similar design features, the models could provide meaningful aggregated effect size estimates across the four studies. Having strong aggregated estimates was important because there were some key differences among the studies that we wished to abstract from. First, the learning conditions that were compared to the RSR condition were different across the four studies. In two studies, the comparison condition was repeated study, in one study it was immediate retrieval, and in the remaining study it was a hybrid condition in which the children had repeated spaced retrieval practice before proceeding to a study-only phase. Second, the RSR conditions in the four studies shared the characteristic of having multiple spaced retrieval trials, but they differed somewhat in the degree of spacing between retrieval trials, and the proportion of study trials that were also included. Third, the novel words in one study represented adjectives, whereas the other studies involved novel words representing nouns. Our main goal was to determine if RSR advantages were robust enough to hold despite these differences, and if so, whether this advantage was influenced by participant group and time. Our previous work allowed us to draw tentative conclusions about these factors, but our analyses did not cross individual study boundaries. We then conducted additional analyses to determine if the results for these main factors of learning condition, participant group, and time held true across studies for each of the test types of word form recall, meaning recall, and recognition.

### The main effects of learning condition, participant group, and time

An especially important set of findings from this study was the overall greater advantage of RSR over the OL conditions. The learning condition effects remained even when the covariates of maternal education and standardized vocabulary tests were included. In addition to these covariates having no bearing on the learning condition effects, we found that they were not even correlated with the children’s final recall and recognition scores. These findings suggest to us that our procedures and outcome measures were capturing a dynamic learning process that is not especially dependent on children’s already-accumulated knowledge. For this reason, they should have wide applicability.

A second encouraging element of the learning condition effects is that the comparison conditions — repeated study and immediate retrieval — were more tightly controlled versions of activities that are generally viewed as helpful to children’s word learning. These activities involve providing children with multiple exposures to new words, and having children repeat new words after hearing them. The children did, in fact, learn words in these comparison conditions; however, this learning did not reach the level seen for RSR.

The advantage of the RSR condition over the comparison conditions was seen at the 1-week testing point as well as during the 5-min test. This 1-week duration of retention suggests that RSR might benefit real-word learning, which depends on words being retained long enough to be incorporated into the lexicon.

It is also clear that the RSR condition provided benefits for both TD children and children with DLD. This type of learning activity, therefore, does not have to be regarded as a strictly remedial procedure. It might be applicable to children with a range of vocabulary skills, as a means of facilitating word learning.

In general, the TD children showed greater accuracy on the final tests than the children with DLD. Given that the TD groups were similar in age to the children with DLD, this is not a surprising finding. Yet, in our previously reported individual studies, we found group differences only in select comparisons. With the strength of the aggregated data with many more observations, the overall differences between the two groups became clear. These differences held even when vocabulary test scores were used as a covariate, so they reflected something more than the children’s already-accumulated vocabulary knowledge.

The children’s test scores did not decline over the 1-week retention period. Along with comporting with the findings from our individual studies, the stability over time is consistent with other investigators’ findings [35].

We should be clear how we interpret the stability found in our data. Specifically, once a threshold was reached where the children could recall a word form or meaning after a second learning session, the children showed an impressive ability to retain that information over 1 week. Note, however, that we refer here to the threshold reached after a *second* day of learning. We did not administer tests at the end of the first learning session. Consolidation processes might have occurred between the first and second day of learning, which allowed the novel words to be incorporated into the children’s lexicons, thereby increasing the likelihood of retention over a longer period.

Even with this qualification in mind, we are encouraged by the fact that the children with DLD were indistinguishable from their peers with typical language development in how well they retained information over 1 week. This suggests that, even at the preschool level, “forgetting” may not be a proper characterization of the vocabulary limitations of children with DLD once these children have shown some initial ability to recall the words.

#### Learning condition interactions with participant group and time

There was no three-way interaction; however, there were 2 two-way interactions involving learning condition. Because interaction effects are generally smaller with the same statistical power as main effects, we reported results from interaction effects with relatively large effect sizes, which revealed several statistically significant simple effects. The RSR advantage held true for both groups of children, but the benefit was greater for the children with DLD. Indeed, the RSR condition showed a smaller difference between the two groups of children (*b*_std_ = 0.19), than did the OL conditions (*b*_std_ = 0.33). Based on conventional alpha levels, the two groups did not differ on the outcome measures for words in the RSR condition. In their study of adults, McGregor et al. [35] also found that retrieval-based practice during the learning period reduced the gap between individuals with DLD and those with typical language skills. In spite of these hopeful signs, we do not believe that our retrieval procedures brought the children with DLD up to the word learning ability of their TD peers. First, although the effect sizes reported here are based on aggregated data, the individual studies contributing to the aggregate were powered to uncover *learning condition effects* — our main focus in the word learning of children with DLD. With larger numbers of children and more words to be learned, group differences might have been seen for the RSR condition, just as we saw for the OL conditions. Second, in some studies, there might have been ceiling effects for the recognition test, which could have constrained the degree of difference between the participant groups in the more favorable (RSR) condition.

The second interaction was that between learning condition and time. The RSR advantage was very clear at both time points. However, the effect size was somewhat smaller at 1 week than at 5 min. Given this slight decrease over the 1-week period, we cannot rule out the possibility that the RSR advantage would diminish over greater lengths of time. Testing over longer stretches of time would be needed to determine if this is true. Even if this were true, we have no indication from the data that any reduction in recall would be greater in the children with DLD. A recent study by McGregor et al. [34] is informative in this regard. Those investigators studied adults with DLD and found retention at 1 month to be comparable to that of their typical language peers.

### Learning condition effects according to test type

Across the four studies, the RSR conditions were associated with higher scores than the OL conditions for all three test types. This is an interesting finding given that the test types differed considerably in the children’s accuracy levels. Specifically, the children’s recognition scores were higher than their meaning recall scores which, in turn, were higher than their scores for word form recall. To respond accurately to a recognition item, the children had to recognize the word form (e.g., /pobɪk/). However, the task required only the ability to associate the word form with the visual referent (e.g., “Where’s the /pobɪk/?”) at least well enough to decide that the visual referent was a better match than the alternatives shown on the laptop screen. The children’s phonological representation of the word form could be quite imprecise for this purpose, given that all of the word forms were phonetically distinct.

For meaning recall, the children had to verbally produce a response, unlike the case for recognition. However, these meanings were familiar words such as “rain” and “birds.” Furthermore, the children could succeed in meaning recall if they matched the familiar word with the *visual referent*; recall of the word form was not required. At the same time, recall of meanings was not a trivial task because we ensured that there were no clues in the pictures as to what each plant or animal liked; these associations were arbitrary and had to be learned as new information.

Although our meaning recall task was not especially difficult for the children, we should not assume that our task was an adequate representation of children’s ability to remember meanings. Only a single meaning had to be learned. We cannot be sure that even an extension of our present task would yield the same results if the children had to remember and associate several different characteristics with the proper referent (what it likes, what it does during the day, what color it changes to over time, etc.). At this point, we can only conclude that on one rather narrow measure of meaning, RSR resulted in greater recall than the comparison learning conditions.

The most difficult test type —word form recall — required the child to produce (novel) words that had never been heard before the study began. Learning these phonological sequences well enough to produce them appeared to be the chief difficulty. This seemed true even though our scoring system allowed for phonetic inaccuracy provided the productions were distinct from plausible alternatives. Note that the children heard each word form three times more often than they heard the corresponding meaning (e.g., “This is a /daɪbo/. It’s a /daɪbo/. A /daɪbo/ likes rocks*.*”) Yet recalling the word form proved more difficult. The children’s accuracy in responding to the word forms in the recognition test suggests that they had an approximate phonological representation of each novel word, but it often was not detailed enough to permit retrieval and production.

The learning condition effect size was especially large for word form (b_std_ = 0.74, *p* = 0.000). Previous studies of children and young adults with DLD have suggested that learning word forms tends to be especially difficult [15, 20, 34]. We should note that in the present study, both groups of children found word form recall to be the most difficult; this can be seen readily in Fig. 3. It is possible, though, that because word form recall was the most difficult for both groups, and the children with DLD had lower scores than their peers, the DLD group might have crossed a clinically significant threshold. That is, it could be that the level of recall shown by the children with DLD on words in the comparison conditions represented a level placing their word learning in significant jeopardy. If this is true, it was helpful to discover in the present study that the RSR condition showed the greatest effect on word form recall. Put differently, repeated spaced retrieval might prove to be a useful approach to assist children with DLD in their weakest area of word learning.

### Repeated spaced retrieval and DLD

What do these findings tell us about children with DLD? These children are clearly less capable word learners than their age mates. However, in many respects they mirror their peers’ learning patterns. First, as with their peers, learning word forms for these children is more difficult than learning single meaning–referent associations and recognizing word form–referent pairings. Second, once these children are capable of recalling a word at 5 min they can retain the word quite well over 1 week’s time. Third, like their counterparts with typical language skills, children with DLD benefit significantly from RSR. In fact, they seem to benefit even more.

The finding that gains occur through RSR suggests something about the “hidden” aspects of word learning in children with DLD. Recently, there has been growing awareness of the importance of considering intake as well as input in the study of children’s language development (e.g., [53]). Note that in our work, the RSR conditions never provided more exposures to the novel words than in the OL conditions, and in one study (study 4), the more retrieval/less study condition involved less exposure than the OL condition. With degree of input controlled, it is intake that seems to have been improved through RSR. We suspect that this occurred in at least two interacting ways. First, consider that studies in memory have found that early retrieval attempts (including pre-tests where the answer is not yet known) seem to prepare learners for the study trial of the same material that follows (e.g., [29]). This has been interpreted as retrieval enhancing subsequent encoding. Words in the repeated study condition obviously allowed for encoding, but this process was not enhanced, much as in conditions in the memory literature where study occurs without a prior test [29].

Second, even when retrieval is correct, when learners have less confidence in their answer, they benefit more from feedback confirming their accuracy than when they are more certain of their answer (e.g., [32]). Note that this factor could be one of the explanations for the advantage that RSR held over immediate retrieval in our study 2. The opportunity to retrieve a word immediately after hearing it should have elevated the children’s expectation that their answer was correct. The feedback that followed, then, would have had less impact on further encoding than instances in which the children ventured a best guess in spaced retrieval that proved to be correct.

There could well be other factors that contributed to the relative advantage of RSR. In fact, one of our studies implicates the effects of some factor independent of feedback, and operating at a point well after encoding is likely to play a major role. In study 4, words in all conditions initially went through a phase that included both immediate retrieval and spaced retrieval with feedback. Then, half the words proceeded to a retrieval-only phase with no feedback while the remaining words proceeded to a study-only phase where, of course, the words and meanings continued to be presented. Recall at the 5-min point and 1 week later proved to be superior for the words that went through the retrieval-only phase. Thus, having more opportunities for retrieval was beneficial even when this occurred later in the learning period without any feedback. Some retrieval accounts in the memory literature (e.g., [54]) hold that as successful retrieval trials accumulate, there is increased precision in accessing the item from memory. Testing the applicability of accounts such as this to children’s novel word learning in future research might shed light on this aspect of our findings.

## Conclusions

Analysis of the aggregated data across our four studies provides reason to be encouraged about the potential benefits of RSR for word learning in children with DLD. In these controlled laboratory studies, different variations of RSR were associated with greater recall than learning conditions that also had merit but yielded results of lower magnitude. Such differences were seen across three different test types. The RSR advantage was still apparent when testing occurred 1 week after the learning sessions had ended. This does not appear to be a transitory phenomenon.

Together, our findings suggest at least two possible directions for future research. More needs to be learned about the precise mechanisms that operate in giving RSR an advantage over other word learning procedures, and whether they function in the same way in children with DLD. A second direction relates to intervention: With further refinement, might RSR be incorporated in treatment procedures designed to assist children with DLD in the learning of new words? Can these procedures be translated to formats (illustrated children’s books in hard copy or electronic form) that are more conducive to application in clinical and educational contexts? We believe the aggregated findings from this study provide a solid basis for pursuing such questions.

## Supplementary Information

**Additional file 1: Analytic Methods. Table 1S.** Experiment Variable Interaction Model Results - pooled over studies and testing type (n = 101, 960 repeated observations). **Table 2S**. Simple Effects for the Condition by Time interaction P pooled Over Studies and Test Type. **Table 3S.** Simple Effects for the Group by Condition interaction for Word Form Recall. **Table 4S.** Simple Effects for the Condition by Time interaction for Word Form Recall. **Table 5S.** Simple Effects for the Group by Time interaction for Word Form Recall. **Table 6S**. Meaning Main Effects Model Results - pooled over studies (*n* = 75, 300 repeated observations). **Table 7S**. Recognition Main Effects Model Results - pooled over studies (*n* = 100, 244 repeated observations).

**Additional file 2: Figure 1S.** Box plots for predicted values by participant group and learning condition. (Computed with Stata v.16.1). RSR = repeated spaced retrieval condition; OL = other (comparison) learning condition; DLD = children with developmental language disorder; TD = children with typical language development.

**Additional file 3: Figure 2S.** Box plots for predicted values by test type and learning condition. (Computed with Stata v.16.1). RSR = repeated spaced retrieval condition; OL = other (comparison) learning condition.

## Data Availability

The datasets used and/or analyzed for this study are available from the corresponding author on reasonable request.

## Abbreviations

CI: Confidence interval
DLD: Children with developmental language disorder
K-ABC2: Kaufman Assessment Battery for Children, Second Edition
OL: Other learning condition
PPVT-4: Peabody Picture Vocabulary Test, Fourth Edition
PTONI: Primary Test of Nonverbal Intelligence
RS: Repeated study
RSR: Repeated spaced retrieval
SPELT-P2: Structured Photographic Expressive Language Test–Preschool 2
