# Supplementary material for: A multi-study examination of the role of repeated spaced retrieval in the word learning of children with developmental language disorder
Source: J Neurodev Disord. 2021 May 15;13:20. doi: 10.1186/s11689-021-09368-z (PMC8126157; doi:10.1186/s11689-021-09368-z)
Supplement: Supplementary file 1 — Additional file 1: Analytic Methods. Table 1S. Experiment Variable Interaction Model Results - pooled over studies and testing type (n = 101, 960 repeated observations). Table 2S. Simple Effects for the Condition by Time interaction P pooled Over Studies and Test Type. Table 3S. Simple Effects for the Group by Condition interaction for Word Form Recall. Table 4S. Simple Effects for the Condition by Time interaction for Word Form Recall. Table 5S. Simple Effects for the Group by Time interaction for Word Form Recall. Table 6S. Meaning Main Effects Model Results - pooled over studies (n = 75, 300 repeated observations). Table 7S. Recognition Main Effects Model Results - pooled over studies (n = 100, 244 repeated observations). [file 11689_2021_9368_MOESM1_ESM.docx]

**Supplementary Materials**

**Analytic Methods**

The general, main effects model, is given in equation (1)

$$\begin{matrix} y_{ij}=\gamma_{00}+\gamma_{01}\text{group}_{j}+\gamma_{10}\text{condition}_{ij}+\gamma_{20}\text{time}_{ij}+\gamma_{02}\text{study2}_{j}+\gamma_{03}\text{study3}_{j}+\gamma_{04}\text{study4}_{j} \\ +\gamma_{30}\text{meaning}_{ij}+\gamma_{40}\text{recognition}_{ij}+\gamma_{05}\text{PPVT}_{j}+\text{ }\gamma_{06}\text{motheduc}_{j}+\gamma_{50}\text{numwords}_{ij} \\ +\mu_{j}+\mu_{10j}\text{condition}_{ij}+\mu_{30j}\text{meaning}_{ij}+\mu_{40j}\text{recognition}_{ij}+r_{ij}\text{ (1)} \end{matrix}$$

Where $y_{ij}$ is the number of correct responses for trial *i* and child *j*. The DLD versus TD group difference is estimated with $\gamma_{01}$, the learning condition difference (RSR versus OL) is estimated with $\gamma_{10}$, and one week versus five minutes post intervention difference is estimated with $\gamma_{20}$. Study differences are estimated with $\gamma_{02}$, $\gamma_{03}$, and $\gamma_{04}$where Study 1 is the reference. Post modeling contrasts are used to compare Studies 2, 3, and 4 with each other. Test type differences are estimated with $\gamma_{30}$and $\gamma_{40}$where word form recall is the reference. Post modeling contrasts are used to compare meaning recall and recognition to one another. The two between-child covariates, PPVT-4 and mother’s education are estimated with $\gamma_{05}$ and $\gamma_{06}$, respectively. The within-child covariate, number of items tested, is estimated with$\gamma_{50}$, The random intercept term,$\mu_{j}$, measures the conditional between-child heterogeneity in the number of correct responses. Three random slopes are included to estimate the conditional between-child variability in the effects of learning condition ($\mu_{10j}$) and test type conditions ($\mu_{30j}$and $\mu_{40j}$) on number of correct responses. The *r_ij_* is the trial-level residual.

The general, main effects model, for within test type outcomes, is given in equation (2)

$$\begin{matrix} y_{ij}=\gamma_{00}+\gamma_{01}\text{group}_{j}+\gamma_{10}\text{condition}_{ij}+\gamma_{20}\text{time}_{ij}+\gamma_{02}\text{study2}_{j}+\gamma_{03}\text{study3}_{j}+\gamma_{04}\text{study4}_{j} \\ +\gamma_{05}\text{PPVT}_{j}+\text{ }\gamma_{06}\text{motheduc}_{j}+\mu_{j}+\mu_{10j}\text{condition}_{ij}+r_{ij}\text{ (2)} \end{matrix}$$

Where $y_{ij}$ is the number of correct responses for trial *i* and child *j* for a specific test type. The DLD versus TD group difference is estimated with $\gamma_{01}$, the learning condition difference (RSR versus OL) is estimated with $\gamma_{10}$, and one week versus five minutes post intervention difference is estimated with $\gamma_{20}$. Study differences are estimated with $\gamma_{02}$, $\gamma_{03}$, and $\gamma_{04}$where Study 1 is the reference. Post modeling contrasts are used to compare Studies 2, 3, and 4 with each other. The two between-child covariates, PPVT-4 and mother’s education are estimated with $\gamma_{05}$ and $\gamma_{06}$, respectively. The random intercept term,$\mu_{j}$, measures the conditional between-child heterogeneity in the number of correct responses. One random slope is included to estimate the conditional between-child variability in the effects of learning condition ($\mu_{10j}$) on number of correct responses. The *r_ij_* is the trial-level residual.

Table 1S. Experiment Variable Interaction Model Results - pooled over studies and testing type (n = 101, 960 repeated observations)

| *fixed effects* | b | | | 95% CI | | | | p-value | | b_std_ | | |
| --- | --- | --- | --- | --- | --- | --- | --- | --- | --- | --- | --- | --- |
| group (DLD vs. TD) | -1.50 | | | -2.61 | | -0.38 | | 0.009 | | | -0.33 | | |
| condition (RR vs. Other) | 1.57 | | | 0.96 | | 2.18 | | 0.000 | | | 0.35 | | |
| time (1wk vs. 5min) | 0.20 | | | -0.18 | | 0.59 | | 0.298 | | | 0.04 | | |
|  |  | | |  | |  | |  | | |  | | |
| study 2 vs. study 1 | -2.90 | | | -4.08 | | -1.72 | | 0.000 | | | -0.64 | | |
| study 3 vs. study 1 | -2.21 | | | -3.39 | | -1.02 | | 0.000 | | | -0.49 | | |
| study 4 vs. study 1 | -0.05 | | | -1.26 | | 1.17 | | 0.940 | | | -0.01 | | |
| study 3 vs. study 2 | 0.69 | | | -0.45 | | 1.83 | | 0.235 | | | 0.15 | | |
| study 4 vs. study 2 | 2.85 | | | 1.54 | | 4.17 | | 0.000 | | | 0.63 | | |
| study 4 vs. study 3 | 2.16 | | | 0.62 | | 3.71 | | 0.006 | | | 0.48 | | |
|  |  | | |  | |  | |  | | |  | | |
| meaning vs. word form | 4.43 | | | 3.84 | | 5.01 | | 0.000 | | | 0.98 | | |
| recognition vs. word form | 5.80 | | | 5.25 | | 6.36 | | 0.000 | | | 1.28 | | |
| meaning vs. recognition | -1.38 | | | -2.16 | | -0.59 | | 0.001 | | | -0.30 | | |
|  |  | | |  | |  | |  | | |  | | |
| 2-way interactions |  | | |  | |  | |  | | |  | | |
| group X condition | 0.65 | | | -0.14 | | 1.44 | | 0.105 | | | 0.14 | | |
| group X time | 0.00 | | | -0.45 | | 0.45 | | 0.991 | | | 0.00 | | |
| condition X time | -0.52 | | | -0.94 | | -0.09 | | 0.018 | | | -0.11 | | |
|  |  | | |  | |  | |  | | |  | | |
| covariates |  | | |  | |  | |  | | |  | | |
| PPVT-4 | -0.02 | | | -0.05 | | 0.02 | | 0.428 | | | 0.00 | | |
| mother's education | 0.00 | | | -0.21 | | 0.22 | | 0.974 | | | 0.00 | | |
| number of words tested | 0.58 | | | 0.44 | | 0.71 | | 0.000 | | | 0.13 | | |
| intercept | 1.04 | | | -4.31 | | 6.39 | | 0.704 | | | -1.38 | | |
|  |  | | |  | |  | |  | | |  | | |
| *random effects* | | σ^2^ | 95% CI | | | |  | |
| condition | | 2.86 | 1.83 | | 4.46 | |  | |  | | |
| meaning | | 5.35 | 3.57 | | 8.02 | |  | |  | | |
| recognition | | 3.31 | 2.09 | | 5.24 | |  | |  | | |
| intercept | | 4.01 | 2.81 | | 5.73 | |  | |  | | |
| Residual | | 2.74 | 2.41 | | 3.12 | |  | |  | | |

*Note*. b_std_ - outcome standardized across studies and testing type; CI = confidence interval; DLD = children with developmental language disorder; TD = children with typical language development; RSR = repeated spaced retrieval; OL = other learning; PPVT-4 = Peabody Picture Vocabulary Test – Fourth Edition.

Table 2S. Simple Effects for the Condition by Time interaction P pooled Over Studies and Test Type

|  | b | 95% CI | | *p*-value | b_std_ |
| --- | --- | --- | --- | --- | --- |
| 1 week versus 5 minutes for RSR condition | -0.31 | -0.70 | 0.07 | 0.111 | -0.07 |
| 1 week versus 5 minutes for OL condition | 0.20 | -0.18 | 0.59 | 0.298 | 0.04 |
|  |  |  |  |  |  |
| RSR versus OL condition at 5 minutes | 1.57 | 0.96 | 2.18 | 0.000 | 0.35 |
| RSR versus OL condition at 1 week | 1.06 | 0.48 | 1.64 | 0.000 | 0.23 |

*Note*. b_std_ = outcome standardized across studies and testing type; CI = confidence interval; DLD = children with developmental language disorder; TD = children with typical language development; RSR = repeated spaced retrieval; OL = other learning.

Table 3S. Simple Effects for the Group by Condition interaction for Word Form Recall

|  | b | 95% CI | | *p*-value | b_std_ |
| --- | --- | --- | --- | --- | --- |
| DLD versus TD in RSR condition | -1.13 | -2.86 | 0.59 | 0.197 | -0.32 |
| DLD versus TD in OL condition | -1.76 | -3.08 | -0.44 | 0.009 | -0.50 |
|  |  |  |  |  |  |
| RSR versus OL condition for DLD group | 3.13 | 2.23 | 4.03 | 0.000 | 0.89 |
| RSR versus OL condition for TD group | 2.50 | 1.61 | 3.39 | 0.000 | 0.71 |

*Note*. b_std_ - outcome standardized across studies WITHIN testing type; CI = confidence interval; DLD = children with developmental language disorder; TD = children with typical language development; RSR = repeated spaced retrieval; OL = other learning.

Table 4S. Simple Effects for the Condition by Time interaction for Word Form Recall

|  | b | 95% CI | | *p*-value | b_std_ |
| --- | --- | --- | --- | --- | --- |
| 1 week versus 5 minutes for RSR condition | -0.32 | -0.75 | 0.11 | 0.145 | -0.09 |
| 1 week versus 5 minutes for OL condition | 0.06 | -0.38 | 0.49 | 0.795 | 0.02 |
|  |  |  |  |  |  |
| RSR versus OL condition at 5 minutes | 2.50 | 1.61 | 3.39 | 0.000 | 0.71 |
| RSR versus OL condition at 1 week | 2.12 | 1.23 | 3.01 | 0.000 | 0.60 |

*Note*. b_std_ - outcome standardized across studies WITHIN testing type; CI = confidence interval; RSR = repeated spaced retrieval; OL = other learning.

Table 5S. Simple Effects for the Group by Time interaction for Word Form Recall

|  | b | 95% CI | | *p*-value | b_std_ |
| --- | --- | --- | --- | --- | --- |
| 1 week versus 5 minutes for DLD group | 0.34 | -0.09 | 0.77 | 0.125 | 0.10 |
| 1 week versus 5 minutes for TD group | 0.06 | -0.38 | 0.49 | 0.795 | 0.02 |
|  |  |  |  |  |  |
| DLD versus TD at 5 minutes | -1.76 | -3.08 | -0.44 | 0.009 | -0.50 |
| DLD versus TD at 1 week | -1.48 | -2.80 | -0.16 | 0.028 | -0.42 |

Note. b_std_ - outcome standardized across studies WITHIN testing type; CI = confidence interval; DLD = children with developmental language disorder; TD = children with typical language development.

Table 6S. Meaning Main Effects Model Results - pooled over studies (n = 75, 300 repeated observations)

|  |  | | | | | | | |
| --- | --- | --- | --- | --- | --- | --- | --- | --- |
|  |  | | | | | | | |
| *fixed effects* | B | 95% CI | | *p*-value | b_std_ | |
| group (DLD vs. TD) | -1.25 | -2.36 | -0.14 | 0.027 | | -0.45 | |
| condition (RSR vs. OL) | 0.71 | 0.30 | 1.12 | 0.001 | | 0.25 | |
| time (1wk vs. 5min) | -0.03 | -0.26 | 0.19 | 0.776 | | -0.01 | |
|  |  |  |  |  | |  | |
| study 2 vs. study 1 | 3.06 | 1.95 | 4.18 | 0.000 | | 1.10 | |
| study 4 vs. study 1 | -1.38 | -2.57 | -0.18 | 0.024 | | -0.49 | |
| study 4 vs. study 2 | -4.44 | -5.46 | -3.42 | 0.000 | | -1.59 | |
|  |  |  |  |  | |  | |
| covariates |  |  |  |  | |  | |
| PPVT-4 | -0.02 | -0.06 | 0.02 | 0.350 | | -0.01 | |
| mother's education | -0.02 | -0.24 | 0.20 | 0.852 | | -0.01 | |
| Intercept | 9.71 | 4.20 | 15.22 |  | |  | |
|  |  |  |  |  | |  | |
| *random effects* | σ^2^ | 95% CI | |  | |  | |
| Condition | 2.37 | 1.46 | 3.85 |  | |  | |
| Intercept | 3.27 | 2.21 | 4.85 |  | |  | |
| Residual | 0.97 | 0.75 | 1.25 |  | |  | |
|  |  |  |  |  | |  | |

*Note*. b_std_ - outcome standardized across studies WITHIN testing type; CI = confidence interval; DLD = developmental language disorder; TD = typically developing; RSR = repeated spaced retrieval; OL = other learning conditions; PPVT-4 = Peabody Picture Vocabulary Test – Fourth Edition.

Table 7S. Recognition Main Effects Model Results - pooled over studies (n = 100, 244 repeated observations)

|  |  | | | | |
| --- | --- | --- | --- | --- | --- |
|  |  | | | | |
| *fixed effects* | b | 95% CI | | *p*-value | b_std_ |
| group (DLD vs. TD) | -1.25 | -2.21 | -0.29 | 0.011 | -0.35 |
| condition (RSR vs. OL) | 0.56 | 0.18 | 0.93 | 0.003 | 0.15 |
| time (1wk vs. 5min) | -0.11 | -0.74 | 0.53 | 0.744 | -0.03 |
|  |  |  |  |  |  |
| study 2 vs. study 1 | -5.30 | -6.39 | -4.21 | 0.000 | -1.47 |
| study 3 vs. study 1 | -2.86 | -3.88 | -1.85 | 0.000 | -0.79 |
| study 4 vs. study 1 | -7.89 | -9.01 | -6.77 | 0.000 | -2.19 |
| study 3 vs. study 2 | 2.44 | 1.46 | 3.42 | 0.000 | 0.68 |
| study 4 vs. study 2 | -2.59 | -3.59 | -1.59 | 0.000 | -0.72 |
| study 4 vs. study 3 | -5.03 | -6.02 | -4.03 | 0.000 | -1.40 |
|  |  |  |  |  |  |
| covariates |  |  |  |  |  |
| PPVT-4 | 0.01 | -0.03 | 0.04 | 0.734 | 0.00 |
| mother's education | -0.05 | -0.24 | 0.14 | 0.604 | -0.01 |
| Intercept | 16.27 | 11.63 | 20.91 |  |  |
|  |  |  |  |  |  |
| *random effects* | σ^2^ | 95% CI | |  |  |
| Condition | 0.37 | 0.01 | 11.65 |  |  |
| Intercept | 2.76 | 1.82 | 4.18 |  |  |
| Residual | 1.97 | 1.35 | 2.87 |  |  |
|  |  |  |  |  |  |

Note. b_std_ - outcome standardized across studies WITHIN testing type; CI = confidence interval; DLD = developmental language disorder; TD = typically developing; RSR = repeated spaced retrieval; OL = other learning conditions; PPVT-4 = Peabody Picture Vocabulary Test – Fourth Edition.
