# Supplementary figures and images for: A multi-study examination of the role of repeated spaced retrieval in the word learning of children with developmental language disorder
Source: J Neurodev Disord. 2021 May 15;13:20. doi: 10.1186/s11689-021-09368-z (PMC8126157; doi:10.1186/s11689-021-09368-z)

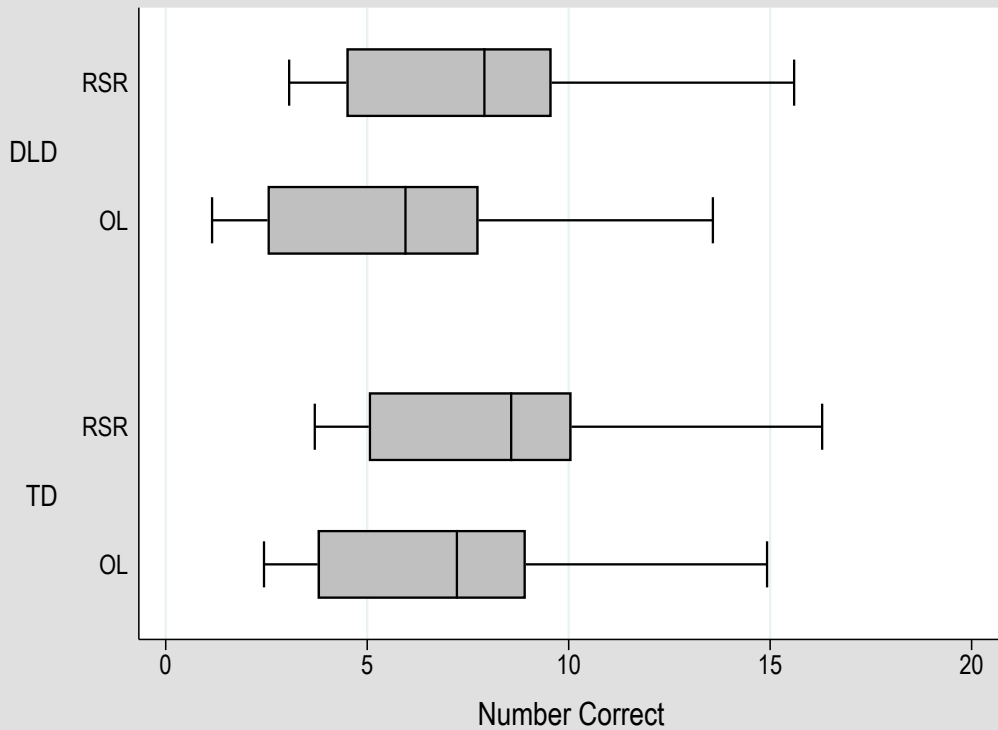

Supplement: Supplementary file 2 — Additional file 2: Figure 1S. Box plots for predicted values by participant group and learning condition. (Computed with Stata v.16.1). RSR = repeated spaced retrieval condition; OL = other (comparison) learning condition; DLD = children with developmental language disorder; TD = children with typical language development. [file 11689_2021_9368_MOESM2_ESM.pdf]

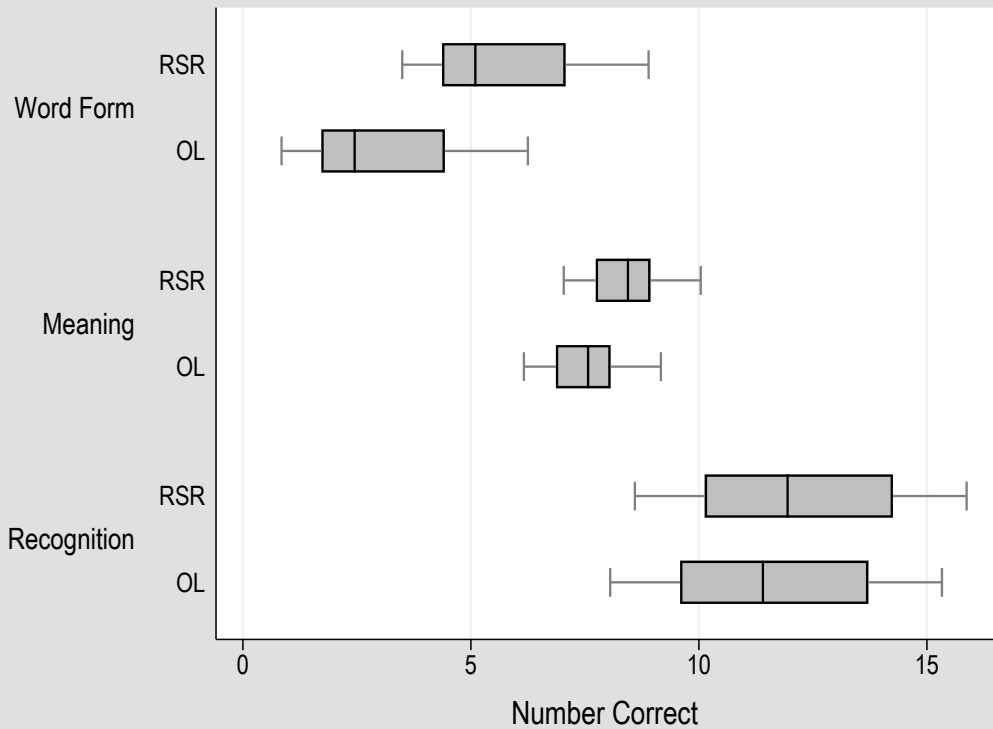

Supplement: Supplementary file 3 — Additional file 3: Figure 2S. Box plots for predicted values by test type and learning condition. (Computed with Stata v.16.1). RSR = repeated spaced retrieval condition; OL = other (comparison) learning condition. [file 11689_2021_9368_MOESM3_ESM.pdf]
